# Development of a Multiplex Amplification System Using Oxford Nanopore Sequencing for STRs and InDels

**DOI:** 10.1155/humu/6687864

**Published:** 2026-04-09

**Authors:** Wei Han, Qingzhen Zhang, Xiaochang Zhang, Mingjie Feng, Hao Xu, Xiaoen Geng, Yixin Wang, Zhe Zhou

**Affiliations:** ^1^ Laboratory of Biotechnology, Bioinformatics Center of AMMS, Beijing, China

**Keywords:** forensic DNA analysis, InDels, kinship testing, nanopore sequencing, STRs

## Abstract

Nanopore sequencing has emerged as a promising technology due to its real‐time data acquisition, portability, and high throughput. However, the genotyping capabilities of the latest flow cell, R10.4.1, for short tandem repeats (STRs) remain insufficiently validated, and its accuracy in typing insertions or deletions (InDels) in earlier versions has been rarely investigated. To bridge these gaps, we developed the NanoID panel, a multiplex amplification system incorporating 29 autosomal STRs, 29 Y‐chromosome STRs, 61 autosomal InDels, 2 Y‐chromosome InDels, and amelogenin. We conducted genotyping on 112 unrelated individual samples and screened 114 loci with 100% accuracy. Subsequently, we evaluated these 114 loci for reproducibility, sensitivity, kinship inference, and species specificity. All loci were consistently and accurately genotyped across triplicate experiments. NanoID achieved an accuracy rate exceeding 99.12% (based on the 2‐out‐of‐3 rule) when the DNA input was ≥ 50 pg. The combined power of discrimination and the cumulative probability of exclusion were 1 − 7.990 × 10^−57^ and 1 − 2.299 × 10^−16^, respectively. For full‐sibling kinship testing, the sensitivity, specificity, and accuracy reached 100% at likelihood ratio (LR) cutoff values of 0.0001 and 10000. Nonhuman samples were clearly distinguishable from human samples. These findings strongly support the NanoID system’s effectiveness for individual identification and full‐sibling kinship analysis using nanopore sequencing.

## 1. Introduction

Catastrophic events such as terrorist attacks [1], air crashes [2], and natural disasters [3] usually result in extensive damage and a high number of casualties, often leaving remains challenging to identify. The difficulty in transporting biological samples to laboratories underscores the need for real‐time, on‐site, and accurate forensic identification [4]. Recently, several integrated rapid DNA systems, such as Quick TargSeq, ANDE, and RapidHIT 200, which utilize capillary electrophoresis (CE) and microfluidic technologies, have been developed. However, significant challenges persist, such as the requirement for long amplified DNA fragments and limited sample processing capacity.

Next‐generation sequencing (NGS), also known as massively parallel sequencing (MPS), is widely utilized in disease research, drug therapy, and molecular diagnostics fields [5–7]. Its forensic applications include individual identification, phenotype estimation, ancestry inference, and kinship analysis [8–10]. Unlike CE, which detects only length‐based polymorphisms [11], MPS provides more detailed data by distinguishing sequence variants of the same length [12]. Additionally, MPS enables simultaneous testing of multiple loci, the design of shorter amplicons, and more effective genotyping of degraded DNA [13]. In addition to short tandem repeats (STRs), MPS can also detect single nucleotide polymorphisms (SNPs) and insertions or deletions (InDels), offering further insights into ancestral and phenotypic traits [14–16].

Since its release in 2014, MinION, developed by Oxford Nanopore Technologies (ONT), has significantly advanced nanopore sequencing technology for research and applications [17]. This sequencing technology passes single‐stranded DNA or RNA through nanopore‐sized protein pores under a constant voltage and records nucleotide sequences from ionic current changes as nucleotides traverse the pore. NGS systems are known for their complex workflows, high initial costs, and bulkiness, which make them difficult to use for on‐site testing [18, 19]. In contrast, MinION, weighing less than 87 g, effectively detects various genetic markers and suits on‐site identification with real‐time data processing capabilities [20].

Numerous studies have explored the use of MinION for typing STRs and SNPs, often noting errors in locus typing. The advanced “strspy” algorithm filters out sequences with low matching scores using a genotype database. When combined with the Promega PowerSeq 46GY system and 30 PCR cycles, this approach achieves accurate genotyping of all loci [21]. Ren et al. employed a self‐developed tool, NASTRA, to genotype data generated by the R9.4.1 flow cell. Of the 27 loci evaluated, 18 were accurately genotyped. Using data from the R10.3 flow cell, 16 of 22 loci were successfully genotyped [22].

Several issues exist with the aforementioned studies. First, while the genotyping capabilities of SNPs on nanopore sequencing have been extensively verified, such is not the case for InDels, another common type of biallelic marker. Second, despite the release of the latest flow cell (R10.4.1), its genotyping capability for STRs remains unvalidated. Third, a mature nanopore‐based method for individual identification has yet to be developed. In this study, we developed a multiplex amplification kit, NanoID, to facilitate the simultaneous detection of 29 autosomal STRs (A‐STRs), 29 Y‐chromosome STRs (Y‐STRs), 61 autosomal InDels (A‐InDels), 2 Y‐chromosome InDels (Y‐InDels), and amelogenin (Amel) using the nanopore sequencing platform. Loci with 100% accuracy were identified with unrelated individual samples, followed by evaluations of their reproducibility, sensitivity, performance in kinship testing, and species specificity to demonstrate its reliability.

## 2. Materials and Methods

### 2.1. Locus Selection and Primer Design

The NanoID panel was designed using Primer Premier 5.0 and MFEprimer [23] to ensure primer specificity, optimal length, and annealing temperature. STRs were sourced from two commercially available kits: the STRtyper‐32G PCR Amplification Kit [24] and SureID PathFinder Plus Kit (Health Gene Technologies, Ningbo, China) [25]. In addition, InDels were selected based on our previous research [26], including the Amel for sex determination. Due to unsuccessful amplification, loci including D7S820, D13S317, PentaE, DYS388, DYS447, DYS449, DYS481, DYS518, DYS527, DYS570, and DYS627 were excluded. Prior to the final exclusion of the 11 loci, we attempted primer redesign by adjusting primer length and binding positions to avoid SNP sites and repetitive sequences in the flanking regions. However, these redesigned and optimized primers still exhibited extremely low amplification efficiency. We therefore excluded these 11 loci from the NanoID to ensure the stability and accuracy of the whole amplification system. Ultimately, the custom‐designed panel comprised 29 A‐STRs, 29 Y‐STRs, 63 InDels, and the Amel. It incorporates 18 of the 20 CODIS core loci (excluding D7S820 and D13S317 due to amplification issues) and compensates with 11 highly discriminatory STRs. Detailed information about these markers is provided in Table S1.

### 2.2. Sample Collection, DNA Extraction, and Quantification

Blood card samples (Nuhigh Biotechnologies, Suzhou, China) were obtained from 112 unrelated individuals (71 males and 41 females) of the Chinese Han population. DNA concentrations were determined using the Qubit 3.0 Fluorometer (Thermo Fisher Scientific, Massachusetts) for quantifying standards 9948 (AGCU ScienTech Incorporation, Wuxi, China). All samples were then diluted to a concentration of 1 ng/*μ*L.

### 2.3. PCR Amplification and Library Construction

For blood card samples, a 2‐mm diameter disc was punched out and added directly to the initial PCR reaction mixture. Each sample was then amplified using the NanoID system via a two‐stage PCR process.

The first PCR was performed to amplify the target fragments. The 30 *μ*L reaction mix consisted of 3.5 *μ*L Enhancer buffer NB (1 N), 2.5 *μ*L Enhancer buffer M, 5 *μ*L primer pool (containing all STR and InDel primers listed in Table S2), 10 *μ*L EM808 polymerase mixture, and 9 *μ*L DNA (or nuclease‐free water for blood card samples). The thermal cycling conditions were as follows: 95°C for 3.5 min; 24 cycles of 98°C for 20 s, 60°C for 4 min; and a final extension at 72°C for 5 min.

After purification with magnetic beads, a second PCR was conducted in a 30 *μ*L volume to enrich the targets. The reaction mix contained 13.5 *μ*L of the purified product, 2.5 *μ*L Enhancer buffer M, 2 *μ*L nuclease‐free water, 2 *μ*L UDI primer, and 10 *μ*L EM808 polymerase mixture. The PCR program was as follows: 95°C for 3 min 30 s; 9 cycles of 98°C for 20 s, 58°C for 1 min, and 72°C for 30 s; followed by 72°C for 5 min.

Following a second purification step, the library concentration was quantified using 1 *μ*L of the sample with the Qubit 3.0 Fluorometer. The quality, purity, and integrity of the libraries were assessed using a Bioptic Qsep 100 Bio‐Fragment Analyzer.

### 2.4. Nanopore Sequencing, NGS, and CE

Half of the PCR products from the preceding step were utilized for library construction using the SQK‐NBD114.96 kit from ONT and the NEBNext Library Prep Kit (New England Biolabs, Beijing, China). Two hundred femtomole of purified PCR product per sample was used for end‐prep, followed by native barcode ligation. Barcoded samples were then pooled for adapter ligation, and a final 20 fmol of the pooled library was loaded onto the flow cell for sequencing. Bovine serum albumin (Invitrogen, California, United States) was added to the flow cell priming mix to improve sequencing efficiency. The prepared flow cells were then sequenced using either the MinION Mk1C or GridION platforms (ONT).

The remaining half of the PCR products were processed through a rolling circle amplification procedure to produce sufficient DNA nanoballs for sequencing on the MGISEQ‐2000RS platform (MGI).

For CE, A‐STRs and Amel were amplified using the STRtyper‐32G PCR Amplification Kit. Y‐STRs were amplified using the SureID PathFinder Plus Kit. Subsequently, the PCR products were analyzed using the 3730XL DNA Analyzer (Thermo Fisher Scientific, Massachusetts).

### 2.5. Sensitivity and Species Specificity

The standard DNA 9948 was diluted to create a series of concentration gradients. Libraries were constructed and sequenced in triplicate using input DNA amounts of 5 ng, 1 ng, 500 pg, 200 pg, 100 pg, and 50 pg, according to the established protocol. To assess species specificity, this method was also applied to a variety of nonhuman species, including cats, chickens, cows, dogs, ducks, *Escherichia coli*, fish, horses, pigs, rabbits, rats, and sheep, using 5 ng of DNA per sample.

### 2.6. Kinship Testing

Blood card samples were collected from 27 anonymous donors representing 8 pedigrees, which included 21 pairs of full siblings (FSs). These kinship samples were combined with 95 unrelated individuals, forming a total of 11,088 pairs of unrelated individuals. All samples were amplified using the NanoID kit and sequenced using the MinION Mk1C platform.

### 2.7. Data Processing and Statistical Analysis

Raw read data generated from ONT were base‐called using Dorado 7.2.13, employing superaccurate modes. STR typing of FASTQ files derived from ONT and MGI sequencing was performed using STRait Razor 3.0 [27]. The software’s default settings established a read depth threshold of 10, and the allele coverage ratio (ACR) was calculated for both heterozygous and homozygous alleles by dividing the lower read count by the highest one, setting a threshold of 0.3. Stutter filters were set at 0.15 for DYS385 and 0.1 for all other loci on the MGISEQ platform and at 0.3 for all loci on the ONT platform. The analysis of InDels was conducted using CLC Genomics Workbench 24 (QIAGEN, Germany) on the original FASTQ files. The software employed an alignment strategy similar to Burrows–Wheeler Aligner (BWA) [28] for identifying InDels, encompassing read trimming, reference mapping, mapping analysis, and mutation detection. Based on the positions of InDels in GRCh38, it generated files containing genotyping results and sequencing depth for each site. For the identification of known variants from mappings, parameters were set to a minimum coverage of 20 and a variant detection frequency threshold of 15%.

Data from CE were analyzed using GeneMapper ID‐X v1.6, with fluorescence intensity values above 100 RFU considered as valid detections.

The STR results from ONT, CE, and MGI sequencing were compared, and InDel typing results were contrasted between ONT and MGI data. The likelihood ratio (LR) for FS and unrelated samples was calculated using Merlin software, consistent with methods described in our previous studies [26]. STR nomenclature adhered to the recommendations of the International Society for Forensic Genetics [29, 30]. GraphPad Prism 9.5 was used for data visualization and additional data analysis.

## 3. Results and Discussion

### 3.1. Sequencing Quality

In this study, nanopore sequencing was conducted using 12 flow cells (R10.4.1), processing a total of 183 samples. Each sequencing run included positive and blank controls. For each positive sample, 122 loci were successfully genotyped. Of the total sequencing data, 93.12% achieved a quality score (*Q*) greater than 9, with peak scores between 15 and 16. Consequently, only high‐quality sequencing data with *Q* > 9 were retained. The data outputs from each flow cell are summarized in Table S3. The sequencing data ranged from 5.96 to 14.02 GB, while high‐quality sequencing data ranged from 4.90 to 12.35 GB, constituting 85.12*%* ± 6.88*%* of the total output. Reads with *Q* > 9 averaged 20.66 ± 5.4 million reads.

A positive correlation was observed between pore activity and the generated data (*R* ^2^ = 0.5798). The flow cell with the lowest pore activity had 878 pores and produced 4,644,170 reads. However, the flow cell that produced the highest volume of data did not have the highest pore activity; 1585 pores were used to generate 11,568,995 usable reads. The number of samples loaded per flow cell could be adjusted based on the pore availability identified during quality control to ensure each sample reached the desired number of reads.

For NanoID, amplicon lengths ranged from 170 to 450 bp, increasing to approximately 270–590 bp after end preparation, native barcode ligation, and adapter ligation. The final sequencing results exhibited three prominent peaks corresponding to the DNA electrophoresis profiles generated by the Qsep 100, serving as a preliminary quality check for this method. This indicated that nanopore sequencing maintains consistency within the library size range of 170–450 bp, allowing uniform passage through the pores to generate sequencing data.

### 3.2. Case‐Type Samples and Locus Selection

We initially validated the MGI typing results against CE. STR typing results for all 112 samples were consistent, confirming the reliability of MGI data as a reference for comparing nanopore sequencing. InDels were compared with those from the CLC Genomics Workbench 24 by analyzing the ONT and MGI sequencing files. Previous studies have shown that errors clustered at specific loci [31]. Therefore, we first filtered error‐prone STRs and InDels in the nanopore sequencing data.

Nanopore genotyping results were summarized in Table S4. Of the 12,393 loci, 12,354 were detected, yielding a detection rate of 99.69%. DYF387S1 had the lowest detection rate (67.61%), while each remaining locus exceeded 98%. Among the 12,354 detected loci, 12,224 were correctly genotyped, achieving an accuracy rate of 98.94%. Eight loci showed accuracy rates below 100%, including four A‐STRs (vWA, D18S51, Penta D, and D8S1132), three Y‐STRs (DYF404S1, DYF387S1, and DYS593), and one InDel (rs34399561). Errors in vWA, D18S51, Penta D, D8S1132, and DYF387S1 were primarily caused by stutters, which resulted in incorrect alleles. DYF404S1 and DYS593 exhibited the same length results as MGI but contained single nucleotide variants. DYF404S1 exhibited a sequencing error (CAC → CTC) in nonrepetitive regions, while DYS593 displayed a T → C mutation at the 15th base in the repeat region, which is a common nanopore sequencing error caused by homopolymer accumulation [32, 33]. The mismatch of rs34399561 was closely related to poor allele balance, with an ACR of 0.42 ± 0.19.

Based on the above genotyping results, error‐prone loci were excluded, retaining 25 A‐STRs, 26 Y‐STRs, 62 InDels, and Amel with 100% accuracy. The sequencing depths for the retained loci in 112 unrelated samples are summarized in Figure 1a,b. Loci with an average depth higher than 10,000× included DYS576, DYS596, DYS458, and rs145635184. Meanwhile, DYS389II had the lowest average depth. Similar observations were noted in the GrandFiler Y‐STR panels [34]. This might be explained by the fact that DYS389II shared a forward primer with DYS389I, leading to reduced primer concentration. Additionally, the shorter amplicon product of DYS389I likely contributed to its preferential amplification.

Figure 1Bar charts display locus depths for all case‐type samples, organized from the lowest to highest, with error lines for (a) STRs and (b) InDels. Horizontal red dashed lines indicate the average locus depths: 2422× for STRs and 2177× for InDels.

**Figure:**
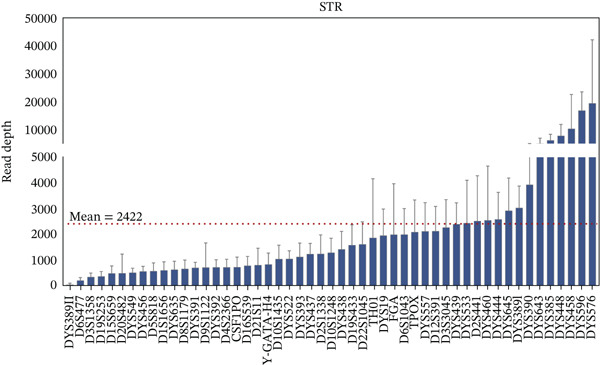
(a)

**Figure:**
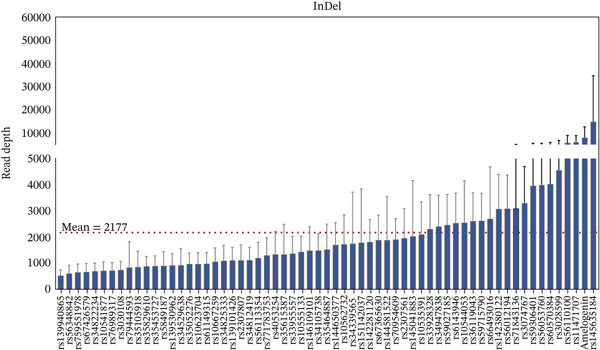
(b)

Figure 2a,b illustrates the ACR for all heterozygotes. The mean ACR for all A‐STRs and DYS385 exceeded 0.7, and the mean ACR for 44 InDels exceeded 0.7. Within InDels, Amel, rs151142037, and rs79444593 had an average ACR < 0.5 with values of 0.17 ± 0.03, 0.41 ± 0.07, and 0.41 ± 0.07, respectively. The results from the MGI sequencing showed no significant allele imbalance (ACR > 0.7) for these three loci, indicating that systematic bias was specific to nanopore sequencing. This phenomenon is likely caused by the inherent sequencing accuracy limitations of the ONT platform, which lead to systematic basecalling errors in one allele strand of these loci, further resulting in a reduced number of valid mapped reads for the affected allele. Homozygotes at D2S1338, D1S1656, FGA, D10S1248, and D19S433 exhibited stuttering, showing a one‐repeat motif (*N* + 1 or *N* − 1) difference. FGA exhibited significant stutter in many NGS kits, primarily due to consecutive adenines in the [AAAG]n repeat structure [35]. Nevertheless, because their ACRs were uniformly below 0.2, they were still identified as homozygotes correctly.

Figure 2Scatter plots display allele coverage ratios (ACRs) of all heterozygotes: (a) STRs and (b) InDels. The red dashed horizontal line indicates a value of 0.5, the yellow solid horizontal line indicates a value of 0.7, and the red short solid line denotes the mean ACR for each locus.

**Figure:**
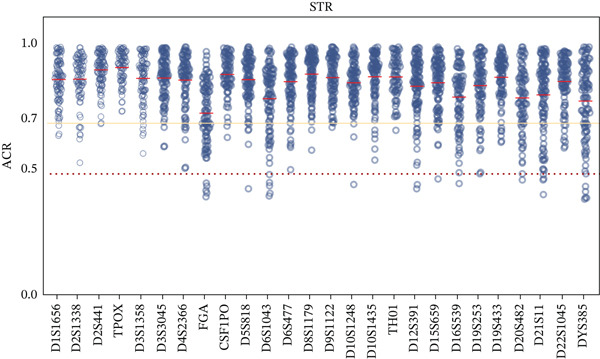
(a)

**Figure:**
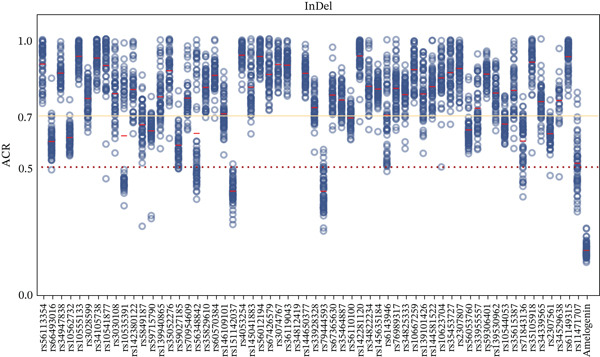
(b)

### 3.3. Reproducibility and Sensitivity

The nanopore genotyping results for standard 9948 were perfectly consistent with the typing results generated by MGI (Table S5). All 114 loci were consistently and accurately genotyped thrice. Three repeated sequencing trials yielded average depths of 2878×, 1804×, and 2623× (Figure 3a) with four loci (DYS458, DYS576, DYS596, and rs145635184) exceeding 10,000× average depth.

Figure 3Read depths of nanopore sequencing for DNA 9948 with inputs of 5 ng, 1 ng, 500 pg, 200 pg, 100 pg, and 50 pg (*n* = 3). (a) Error‐barred scatter plots illustrate variability of read depths for A‐STRs, Y‐STRs, and InDels across different DNA input levels; A‐STRs are represented by deep blue circles, Y‐STRs by light blue squares, and InDels by green hexagons. (b) Box‐and‐whisker plots depict the distribution of read depths for each locus with different DNA inputs. Actual locus depths are indicated by light blue dots.

**Figure:**
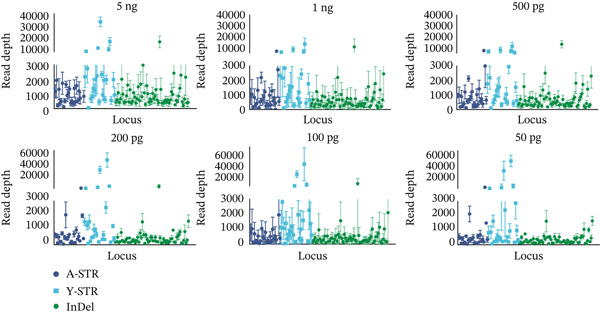
(a)

**Figure:**
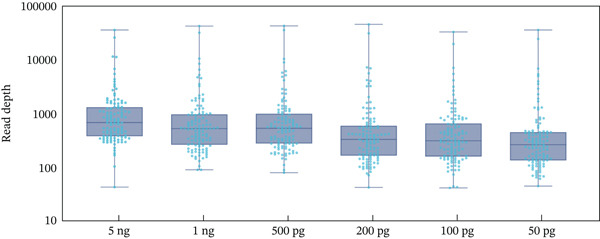
(b)

Allele dropout (ADO) was flagged when the read proportion of the minor allele fell below 0.15. Loci with a total read depth below 20 were classified as having low depth of coverage (LDO), rendering genotype calls unreliable. The increase in ADO and LDO at lower DNA inputs is consistent with stochastic effects in low‐template PCR [36].

The heterozygote balance was represented by ACR, as illustrated in Figure 4a. Of the 114 loci analyzed in DNA 9948, 53 were heterozygous. This group comprised 20 A‐STRs, 1 Y‐STR (DYS385), and 15 InDel loci, with an ACR exceeding 0.7 three times. However, ACRs for Amel, rs66493016, rs5902718, rs79444593, and rs11471707 were consistently below 0.5, specifically averaging 0.17 ± 0.01, 0.47 ± 0.03, 0.47 ± 0.01, 0.42 ± 0.06, and 0.24 ± 0.03, respectively.

Figure 4Allele coverage ratios (ACRs) of nanopore sequencing for DNA 9948 were evaluated with input amounts of 5 ng, 1 ng, 500 pg, 200 pg, 100 pg, and 50 pg (*n* = 3). (a) Error‐barred scatter plots demonstrate the variability in ACRs for heterozygotes in A‐STRs, Y‐STRs, and InDels, as well as for all homozygotes, across different DNA input levels. A‐STRs are represented by deep blue circles, Y‐STRs by light blue squares, InDels by green hexagons, and homozygotes by red triangles. (b) Box‐and‐whisker plots illustrate ACRs for all heterozygotes across varying DNA inputs. Actual locus ACRs are indicated by light blue dots.

**Figure:**
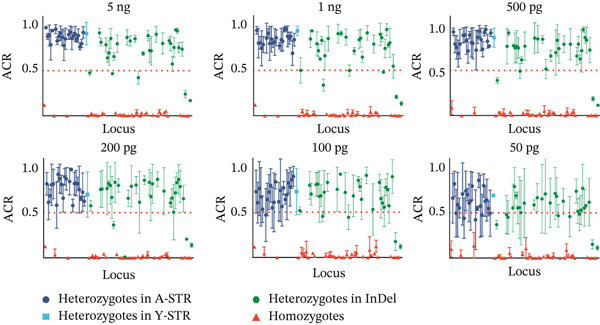
(a)

**Figure:**
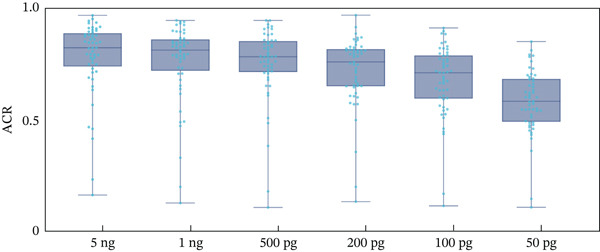
(b)

In DNA 9948, only one homozygous locus, D2S1338, exhibited an ACR > 0.1, where a stutter peak appeared with one less repeat than the true allele. Nevertheless, it remained distinguishable from the heterozygote. The ForenSeq DNA Signature Prep Kit reported that D2S1338 may exhibit stutters or sequencing errors, likely due to nucleotide misincorporations during amplification [37].

Sensitivity was validated using varying amounts of DNA 9948: 5 ng, 1 ng, 500 pg, 200 pg, 100 pg, and 50 pg, with each level repeated thrice. As depicted in Figure 3a,b, lower DNA input levels led to a reduction in overall read depths, consistent with findings from numerous NGS kits [38, 39]. However, contrary to findings from a prior study validating a microhaplotype kit using nanopore sequencing [36], the depth at 5 ng was lower than that at 1 ng, possibly due to the quality and lifespan of the flow cells. When the DNA input was reduced to 200 pg, DYS389II, D6S477 and rs34529638 had average depths of less than 100. Further input reduction to 100 pg and 50 pg resulted in 4 and 8 loci, respectively, with an average depth of less than 100. As the DNA input decreased, the variability in depths increased, and some loci exhibited preferential amplification. Interlocus balance was calculated using the ratio of locus reads divided by the mean coverage. Of the 15 loci showing a relative increase in depths from 5 ng to 50 pg, FGA, DYS458, and DYS576 had ratios exceeding 1.

Reductions and fluctuations in ACRs were more pronounced than those of depths, as shown in Figure 4a,b. At 5 ng DNA input, 5 loci had an average ACR < 0.5, increasing to 12 loci at 50 pg. The number of loci with an ACR > 0.7 decreased from 37 at 5 ng to only 2 at 50 pg. In homozygotes, the proportion of stutter peaks increased for D2S1338 and D16S539 at lower DNA inputs. Additionally, rs10535391, rs60570384, and rs144581522 showed ACRs greater than 0.1.

As shown in Figures S1 and S2, all loci were detected (100%, 117/117) with DNA inputs of 200 pg or higher. At 500 pg, rs11471707 exhibited ADO, potentially due to pre‐existing allelic bias (ACR = 0.24 at 5 ng). At 100 pg, LDOs occurred for D6S447, rs144581522, and rs34529638, while ADOs were observed for D12S391 and rs11471707 (twice), and an allele drop‐in (ADI) was seen for rs3028599. All loci were detected at 50 pg (351/351). However, allele loss was observed: D20S482 lost a long allele, while D3S3045, D4S2366, TPOX, D19S253, D21S11, and D8S1179 lost short alleles. The loss of short alleles contrasts with the typical preferential loss of long alleles observed in NGS [36]. For InDels, rs70954609 and rs11471707 showed ADOs, and rs60570384 showed an ADI.

These results demonstrated that the NanoID kit achieved a detection rate of 100% with a DNA input above 50 pg, reaching an accuracy rate of 96.76%, despite some instances of ADOs, ADIs, or LDOs (Figure S3). The sensitivity performance is comparable to that of NGS panels [34, 40]. Considering that forensic practice often requires two concordant results for verification, the panel successfully genotyped 113 out of 114 loci (excluding the problematic rs11471707) at inputs ≥ 50 pg. Primer sequences for the loci prone to ADO/ADI will be further optimized to improve low‐template performance.

### 3.4. Kinship Testing

**Figure 5:**
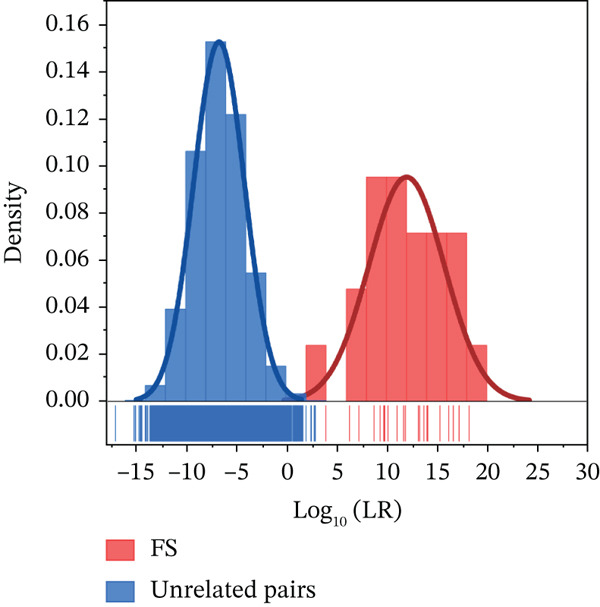
Log_10_LR distribution of full siblings (FSs) and unrelated pairs using NanoID. The red rectangle represents FS, while the blue rectangle represents unrelated pairs.

As shown in Figure 5, using log_10_LR cutoff values of −4 and 4 [26], among 11,088 pairs of unrelated individuals, 9466 pairs had log_10_LR ≤ −4, and 1622 pairs had log_10_LR values in the range of (−4, 4), classifying them as inconclusive. For the 21 pairs of FS, 20 pairs showed log_10_LR ≥ 4, whereas 1 pair showed log_10_LR in the range of (−4, 4), which was categorized as inconclusiveness. Although 14.61% of the samples did not provide a definitive conclusion, the sensitivity, specificity, and accuracy remained at 100% (Table 1). This indicates that the NanoID panel is sufficiently powerful for FS kinship testing.

**Table 1:** System power of NanoID in full‐sibling kinship testing.

Relationship	Threshold 1 (log_10_LR)	Threshold 2 (log_10_LR)	Sensitivity (%)	Specificity (%)	Error rate (%)	Inconclusiveness (%)	Effectiveness^a^ (%)	Accuracy (%)
Full sibling/unrelated	4	−4	100	100	0	14.61	85.39	100

^a^Effectiveness is defined as accuracy minus the inconclusiveness rate and error rate [41] (effectiveness = accuracy − the inconclusiveness rate − error rate).

### 3.5. Species Specificity

**Figure 6:**
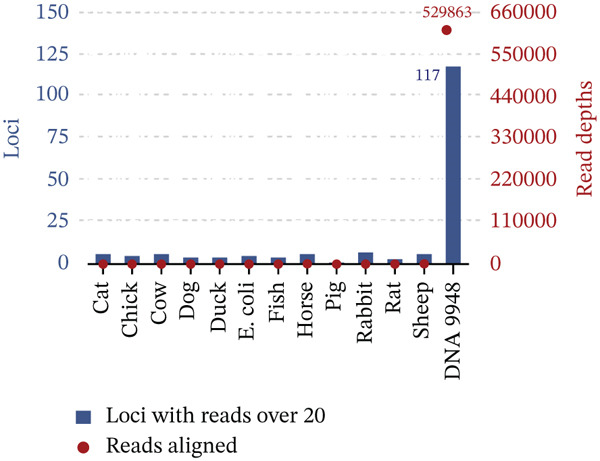
Bar scatter plots represent loci detected in nonhuman and *Homo sapiens* samples, along with their read depths. Blue rectangles on the left *Y*‐axis represent the number of loci where the depth exceeds 20. Red dots on the right *Y*‐axis indicate the read depths for nonhuman samples.

To assess species specificity, we tested 12 nonhuman samples: cat, chicken, cow, dog, duck, *E. coli*, fish, horse, pig, rabbit, rat, and sheep. We set the *Q*‐value threshold to nine, retaining only sequences with *Q* > 9. Ninety‐four percent of the sequences passed this filtering step (Table S6). Using STRait Razor v3.0 and CLC Genomics Workbench 24 for locus calling, we found that 41.06% (611,379 of 1,488,909) of reads from *Homo sapiens* samples mapped to the human reference genome. In contrast, the percentage of aligned reads of species samples was relatively low at 7.78*%* ± 3.92*%*. The loci that exceeded the interpretation thresholds and their aligned reads are shown in Figure 6. Among nonhuman samples, 1–7 loci surpassed the thresholds, with alignment reads ranging from 110× to 1033×, likely due to DNA random amplification. This was significantly different from the standard sample, which detected 117 loci. Our results indicate that the NanoID kit effectively distinguishes between human and nonhuman samples.

## 4. Conclusion

Previous research has focused on improving the accuracy of STR detection through a variety of algorithmic approaches. Notable among these are the BWA [28], minimap2 [42] for aligning long fragments, STRspy [21] which relies on reference databases, and DeepRepeat [43], an innovative method that employs red–green–blue channel analysis for STR genotyping. These techniques have expanded the application of nanopore sequencing in forensic science. In our study, we developed a panel with an average fragment size of 300 bp, adapted for the nanopore sequencing platform. For the analysis of STRs and InDels, we utilized STRait Razor v3.0 and CLC Genomics Workbench 24, tools typically used in NGS workflows. The precision of nanopore sequencing has significantly improved, and with the appropriate choice of analytical methods, even libraries comprising shorter fragments can achieve satisfactory outcomes on Oxford Nanopore systems.

Studies have highlighted the challenges faced by nanopore sequencing technology, particularly in accurately resolving homopolymer regions [21, 31], as evidenced by typing errors observed in loci such as DYF404S1 and DYS593 in our study. To mitigate these issues, researchers have carefully selected STRs that are more amenable to nanopore sequencing, thereby reducing significant sequencing bias [44, 45]. In our research, we successfully genotyped 25 A‐STRs from 112 unrelated individual samples, outperforming recent literature where only 18 out of 27 A‐STRs were accurately genotyped from 80 samples [22]. By employing suitable detection methods for analyzing data generated from the R10.4.1 flow cell, we achieved accurate genotyping of complex loci, including [GGAA]n [GGAG]n [AAAG]n [AGAA]n [AAAA]n [GAAA]n within the FGA and [ATT]n ACT [ATT]2 in D22S1045, which have been previously recognized as challenging. As algorithms and sequencing accuracy continue to improve, it is anticipated that an even broader range of STRs will be reliably typed.

InDels, representing biallelic genetic markers in DNA, exhibit distinct polymorphisms characterized by low mutation rates and small amplicon sizes, thereby addressing some of the limitations associated with STRs and SNPs [44]. NGS has demonstrated a relatively high level of allele balance and accurate typing for InDels. Our study validated the use of InDels in nanopore sequencing as a supplementary tool for forensic genetic applications.

For forensic STR and InDel genotyping, the ONT sequencing platform delivers distinct advantages over the two dominant benchmark technologies in the field: widely adopted CE and NGS sequencing platforms. (1) In terms of throughput, the ONT platform bypasses the inherent fluorescence channel limitations of CE [46], enabling simultaneous profiling of hundreds of genetic markers in a single reaction, with flexible sample loading that accommodates both single‐case and high‐throughput batch forensic testing. (2) Portability: The MinION sequencer weighs only 87 g, which is significantly lighter than conventional forensic genetic analysis platforms, making it ideally suited for on‐site deployment. (3) Speed: The library quantification workflow for the ONT platform only requires fluorometric quantification via the Qubit system, which saves substantial hands‐on time compared with the qPCR‐based quantification required for NGS sequencing. Furthermore, it delivers reportable genotyping results within 1 h of sequencing initiation, with a full end‐to‐end workflow completed within 8 h, compared with 2–3 days for NGS systems [47]. (4) Accuracy: The raw single‐base accuracy of ONT reads is consistently > 99% (Q20), while NGS platforms achieve a raw single‐base accuracy of > 99.9% (Q30). However, using our established method, all selected STR and InDel loci in standard input DNA samples were accurately genotyped, reaching 100% genotyping concordance with reference results. (5) Cost: The upfront capital investment for the ONT platform is lower than that for both CE and NGS sequencing systems; while per‐sample reagent costs are higher compared with mature commercial CE kits, its flexible loading protocol avoids the waste of flow cell throughput inherent to small‐batch runs on NGS systems [48]. Nevertheless, nanopore sequencing still faces several challenges, such as the requirement for high‐quality DNA templates and robust on‐site data analysis capabilities. Our method provides an accessible tool for forensic DNA identification serving in mass disaster scenes. As nanopore sequencing technology progresses and data analysis algorithms are optimized, this technology will play an increasingly important role in DNA testing [49].

The NanoID shows strong practical adaptability for on‐site forensic detection, as it allows direct amplification without complex DNA extraction and is compatible with the portable MinION platform. However, the current workflow still relies on thermal cyclers, magnetic racks, and pipetting equipment and also requires a relatively high‐performance laptop/desktop for sequencing data analysis. For future optimization, we will integrate all required components into a single compact, portable module to build a fully integrated on‐site detection system.

To summarize, we developed and validated NanoID, which included 29 A‐STRs, 29 Y‐STRs, 61 A‐InDels, 2 Y‐InDels, and Amel, facilitating both individual identification and FS kinship analysis. The combined power of discrimination was 1 − 7.990 × 10^−57^, and the cumulative probability of exclusion was 1 − 2.299 × 10^−16^. We also performed methodological evaluations on panel’s accuracy, reproducibility, sensitivity, species specificity, and performance in kinship testing. The results demonstrate that we have developed a nanopore sequencing–based method, providing a reliable solution that expands the application of nanopore sequencing in forensic genetics.

## Author Contributions

Conceptualization and methodology: Z.Z. and Q.Z. Investigation: W.H., M.F., and X.G. Formal analysis: W.H., X.Z., H.X., and Y.W. Writing—original draft: W.H., Q.Z, and Z.Z. W.H. and Q.Z. have contributed equally to this work.

## Funding

This work received funding from the National Natural Science Foundation of China (Grant No. 82402186).

## Disclosure

All authors read and approved the final draft of the manuscript.

## Ethics Statement

All experiments involving human participants in this study were performed in accordance with the ethical standards of the Institutional and National Research Committee.

## Conflicts of Interest

The authors declare no conflicts of interest.

## Supporting Information

Additional supporting information can be found online in the Supporting Information section.

## Supporting information


**Supporting Information 1** Table S1: Information on the selection loci of NanoID. Table S2: Primer information of NanoID. Table S3: Run and software analysis summary of the data produced by each flow cell. Table S4: Genotyping results of all case‐type samples. Table S5: Length‐based and sequencing‐based genotyping results of 9948. Table S6: Run and software analysis results of nonhuman and *Homo sapiens* samples.

**Supporting Information 2** Figure S1: Raster plots represent STR genotype consistency of genotyping results from MGI systems across three replicates with input amounts of DNA 9948 at 5 ng, 1 ng, 500 pg, 200 pg, 100 pg, and 50 pg. Figure S2: Raster plots represent InDel genotype consistency of genotyping results from the MGI systems across three replicates with input amounts of 9948 at 5 ng, 1 ng, 500 pg, 200 pg, 100 pg, and 50 pg. Figure S3: Bar plots illustrate the consistency of genotyping results from MGI systems across three replicates with input amounts of DNA 9948 at 5 ng, 1 ng, 500 pg, 200 pg, 100 pg, and 50 pg.

## Data Availability

The datasets generated during and/or analyzed during the current study are available from the corresponding author on reasonable request.
