# Supplementary material for: Development of a Multiplex Amplification System Using Oxford Nanopore Sequencing for STRs and InDels
Source: Hum Mutat. 2026 Apr 9;2026:6687864. doi: 10.1155/humu/6687864 (PMC13062922; doi:10.1155/humu/6687864)

Suppl. Figure 1: Raster plots represent STR genotype consistency of genotyping results from MGI systems across three replicates with input amounts of DNA 9948 at 5 ng, 1 ng, 500 pg, 200 pg, 100 pg, and 50 pg. In these charts, blue denotes correct genotyping, red denotes error genotyping, and yellow indicates locus drop-out. Amelogenin is included in STRs for image balancing.

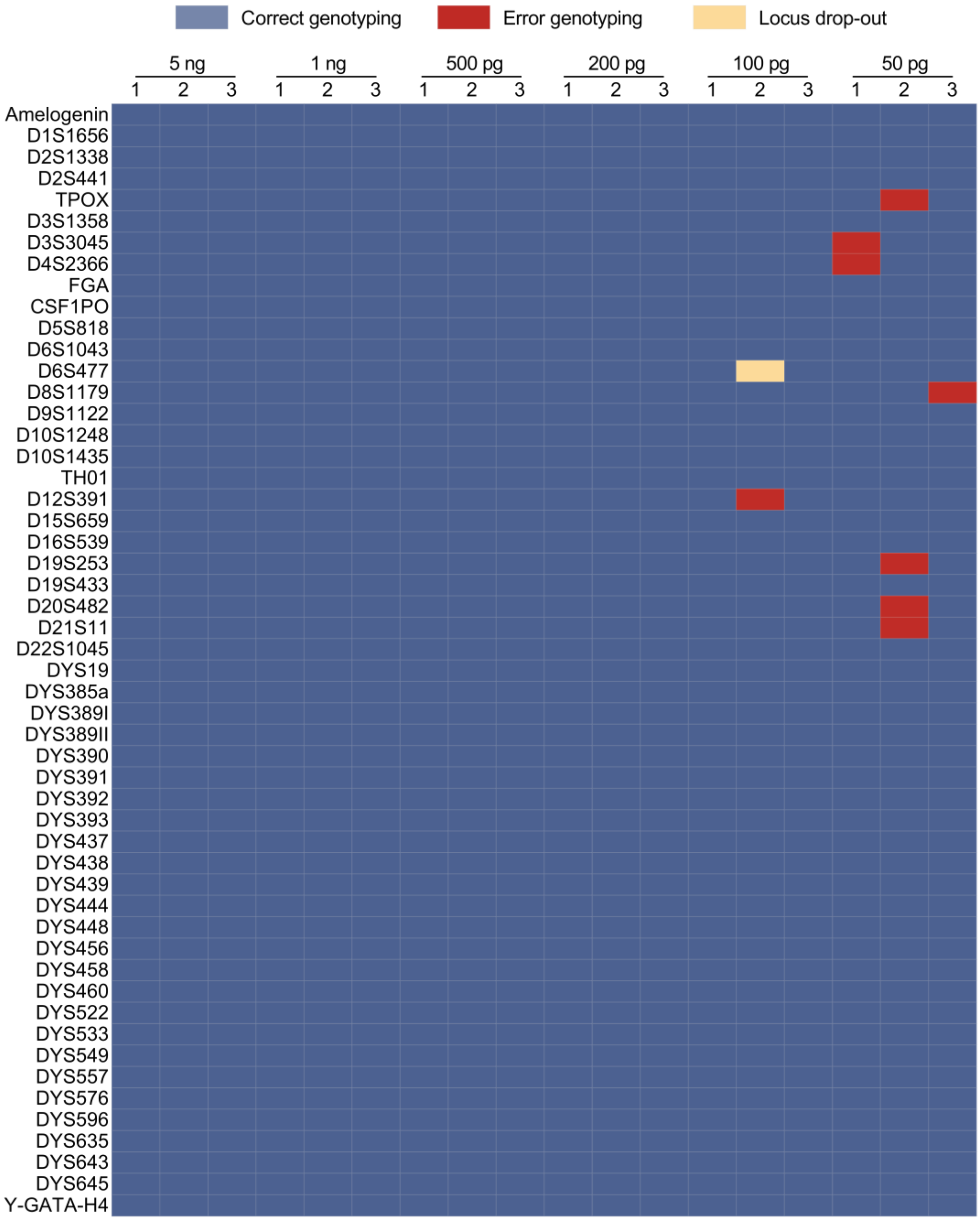

Suppl. Figure 2: Raster plots represent InDel genotype consistency of genotyping results from the MGI systems across three replicates with input amounts of 9948 at 5 ng, 1 ng, 500 pg, 200 pg, 100 pg, and 50 pg. In these charts, blue denotes correct genotyping, red denotes error genotyping, and yellow indicates locus

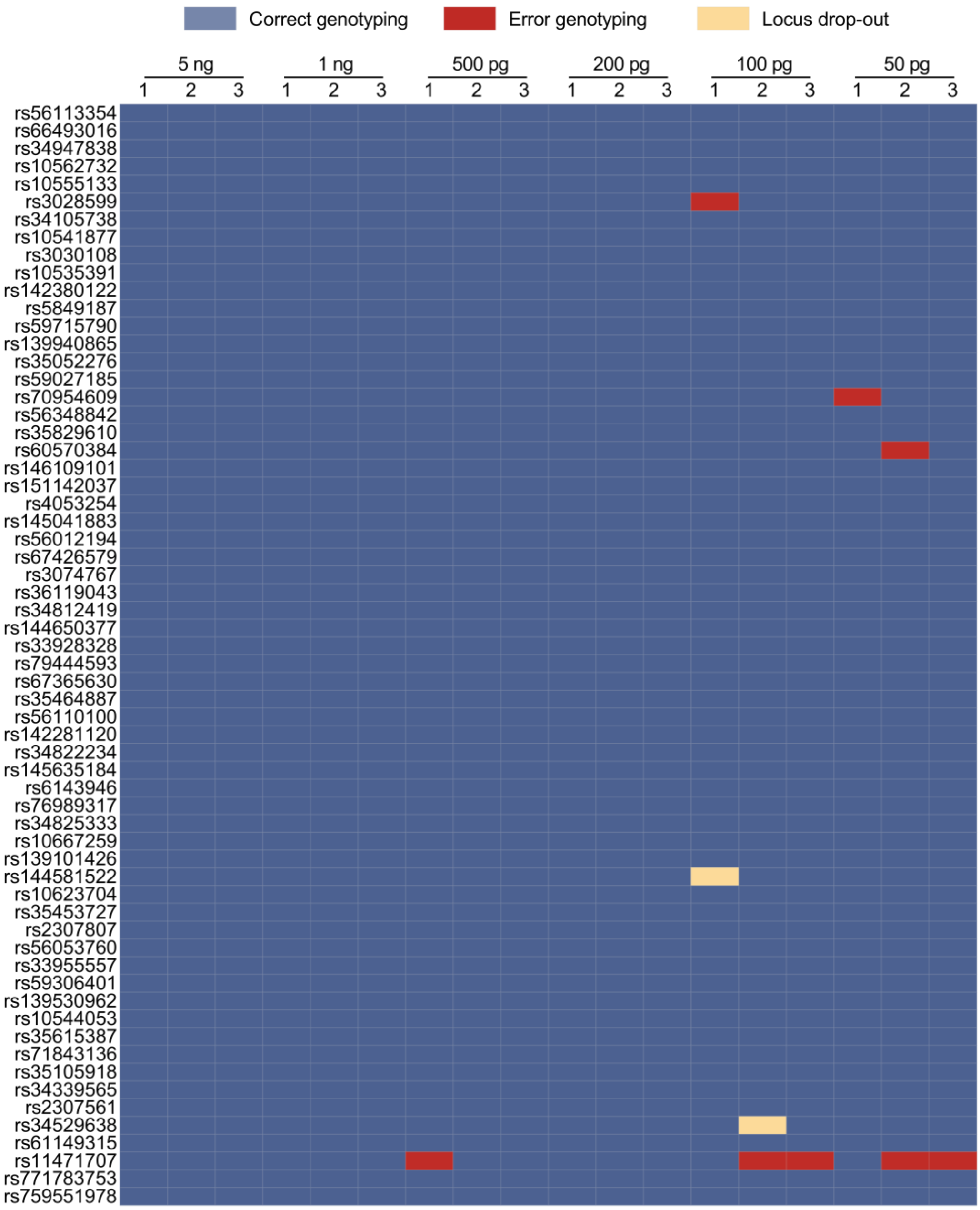

Suppl. Figure 3: Bar plots illustrate the consistency of genotyping results from MGI systems across three replicates with input amounts of DNA 9948 at 5 ng, 1 ng, 500 pg, 200 pg, 100 pg, and 50 pg. Blue, red, and yellow bars denote correct genotyping, erroneous genotyping, and locus drop-out, respectively.

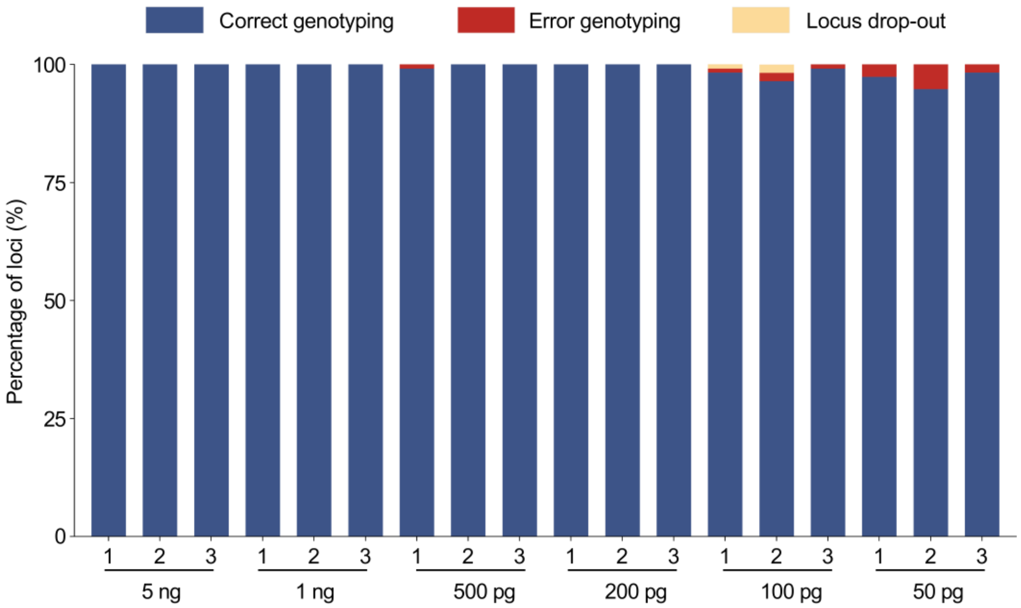

Supplement: Supplementary file 2 — Supporting Information 2 Figure S1: Raster plots represent STR genotype consistency of genotyping results from MGI systems across three replicates with input amounts of DNA 9948 at 5 ng, 1 ng, 500 pg, 200 pg, 100 pg, and 50 pg. Figure S2: Raster plots represent InDel genotype consistency of genotyping results from the MGI systems across three replicates with input amounts of 9948 at 5 ng, 1 ng, 500 pg, 200 pg, 100 pg, and 50 pg. Figure S3: Bar plots illustrate the consistency of genotyping results from MGI systems across three replicates with input amounts of DNA 9948 at 5 ng, 1 ng, 500 pg, 200 pg, 100 pg, and 50 pg. [file HUMU-2026-6687864-s002.pdf]
